# Environmental and Cultivation Effects on Growth and Phytochemical Profiles of Chicory (*Cichorium intybus* L.) in Soil, Hydroponics, and Aquaponics

**DOI:** 10.3390/plants15060974

**Published:** 2026-03-21

**Authors:** Lorenzo Maria Curci, Sara Carrozzo, Gabriele Pecatelli, Teodoro Semeraro, Cosimo Tafuro, Marcello Salvatore Lenucci, Monica De Caroli

**Affiliations:** 1 Department of Biological and Environmental Sciences and Technologies, University of Salento, Via Monteroni 165, 73100 Lecce, Italy; lorenzomaria.curci@unisalento.it (L.M.C.); sara.carrozzo@unisalento.it (S.C.); marcello.lenucci@unisalento.it (M.S.L.); 2 NBFC National Biodiversity Future Center, 90133 Palermo, Italy; 3 Department of Biology and Biotechnologies “Charles Darwin”, Sapienza University of Rome, 00185 Rome, Italy; gabriele.pecatelli@uniroma1.it; 4 Research Institute on Terrestrial Ecosystems (IRET-URT Lecce), National Research Council of Italy (CNR), Campus Ecotekne, 73100 Lecce, Italy; teodoro.semeraro@cnr.it; 5 Department of Research, Development and Innovation, RI Group S.P.A., Via Surbo 38, Trepuzzi, 73019 Lecce, Italy; cosimo.tafuro@rigroup.it

**Keywords:** *Cichorium intybus* L., soil-based cultivation, soilless cultivation, hydroponics, aquaponics, controlled environment, greenhouse, sustainable agriculture

## Abstract

The increasing demand for sustainable food production has intensified interest in controlled-environment agriculture and soilless cultivation systems. This study evaluated the performance of local chicory (*Cichorium intybus* L., cultivar “Otrantina”) grown for 45 days in soil, hydroponics, and decoupled aquaponics under two different environments: a fully controlled growth chamber and a naturally variable greenhouse. Morphological, anatomical, biochemical, and physiological traits were analyzed to assess the combined influence of growth environment and cultivation system on plant development and nutritional quality. Across all parameters, the growth environment emerged as the main driver of plant performance. Greenhouse-grown plants exhibited greater leaf expansion, enhanced mesophyll and vascular development, and higher fresh and dry biomass than those cultivated in the growth chamber. Within each environment, hydroponics consistently supported vigorous growth, whereas aquaponics produced smaller leaves and pronounced root elongation, likely reflecting nutrient and pH instability in the decoupled system. Biochemical analyses revealed system-specific adaptive responses. Soilless cultivation promoted higher lipid accumulation and, under growth chamber conditions, increased protein content. Aquaponically grown plants, particularly in the greenhouse, accumulated elevated levels of soluble sugars and phenolic antioxidants, consistent with stress-related metabolic activation. In contrast, soil-grown plants displayed the highest flavonoid concentrations, suggesting a prominent role of rhizosphere–microbiome interactions in modulating secondary metabolism. Overall, these results indicate that, under the tested conditions, environmental control exerts a stronger influence than cultivation systems on chicory growth and metabolism. Hydroponics proved to be the most efficient system for biomass production, whereas aquaponics requires improved nutrient management to ensure stable growth and quality. The distinct metabolic profiles associated with each cultivation system highlight opportunities to tailor chicory nutraceutical traits within sustainable controlled-environment agriculture.

## 1. Introduction

Agriculture is at the center of intense public and scientific debate, as its future is increasingly challenged by demographic, environmental, and socioeconomic pressures. In recent years, scientific output on agricultural systems has grown exponentially, reflecting efforts to reconcile productivity with sustainability and to reassess the balance between conventional and unconventional farming practices [1]. This renewed attention is driven by the convergence of three major factors. First, rapid global population growth and rising food demand place unprecedented pressure on agricultural systems to ensure food security while maintaining resource efficiency [2]. Second, agriculture both contributes to and is profoundly affected by climate change, necessitating the development of resilient and environmentally sustainable production strategies capable of withstanding increasing climatic variability [3,4]. Third, agricultural production is embedded within complex local, regional, and global socioeconomic dynamics, encompassing food cultivation, processing, distribution, and consumption. Collectively, these systems constitute the largest human economic activity, exceeding other major industries such as computing, pharmaceuticals, energy, and finance in scale and societal relevance [5].

Plants exhibit species-specific physiological and metabolic responses to environmental cues, which are increasingly disrupted by climate-driven stressors [6]. In this context, soilless cultivation within protected environments has emerged as a promising approach to mitigate these impacts [7]. Protected-environment agriculture, encompassing greenhouses and growth chambers, enables precise regulation of temperature, humidity, CO_2_ concentration, and light. Greenhouses integrate natural radiation with technologies such as heating, cooling, shading, and supplemental LED lighting, whereas growth chambers provide fully artificial and highly controlled conditions, allowing year-round production and targeted manipulation of plant metabolic pathways [8,9]. Advances in sensor networks, automation, and decision-support systems further enhance resource-use efficiency and crop performance by enabling continuous monitoring and dynamic control of environmental parameters [10]. Practices such as CO_2_ enrichment, selective shading, and customized light spectra have been shown to improve photosynthesis, biomass accumulation, and phytochemical synthesis, while energy-efficient technologies reduce the environmental footprint of protected cultivation systems [11,12,13].

Protected cultivation is closely associated with soilless production systems, which allow precise nutrient management and improved crop uniformity and quality. Hydroponic systems supply essential nutrients directly in aqueous solution, enabling strict control of pH and mineral composition, accelerated growth rates, vertical production, and enhanced accumulation of bioactive compounds [14,15]. Aquaponics further integrates aquaculture and hydroponics into a closed-loop system in which nutrients derived from fish effluents sustain plant growth, while plants contribute to water purification. This synergistic approach reduces fertilizer inputs and environmental impacts while improving crop yield and nutritional quality [16,17]. Both hydroponic and aquaponic systems increasingly rely on sensor-based monitoring and AI-assisted automation to maintain optimal water chemistry, dissolved oxygen, and nutrient balance, thereby enhancing system stability and operational efficiency [18]. Despite ongoing challenges related to nutrient variability and biofilter management, aquaponic systems have demonstrated significant advantages over conventional cultivation in terms of yield and product quality [19,20].

As climate change intensifies exposure to heat stress, irregular radiation patterns, and water scarcity, controlled-environment agriculture represents a strategic tool for both mitigation and adaptation. Comparative evaluations of soil-based, hydroponic, and aquaponic systems under distinct environmental conditions are therefore essential to identify cultivation strategies capable of stabilizing yield, quality, and functional traits under fluctuating climatic scenarios.

Within this framework, the present study compares soil, hydroponic, and decoupled aquaponic cultivation systems under greenhouse and growth-chamber conditions using the local *Cichorium intybus* L. “Otrantina” variety. Following germination, plants were grown for 45 days, and growth performance, bioactive compound accumulation, and overall plant health were assessed. Chicory is widely cultivated in southern Italy and is valued for its adaptability, morphological diversity, and high concentrations of inulin, phenolic acids, flavonoids, and sesquiterpene lactones, compounds associated with antioxidant, anti-inflammatory, prebiotic, and hepatoprotective properties [21,22]. These characteristics make chicory particularly well suited to soilless cultivation under controlled environments, supporting sustainable production systems, conservation of local agrobiodiversity, and enhanced nutritional and functional quality [23].

## 2. Results

### 2.1. Chicory Plant Morphology

**Figure 1:**
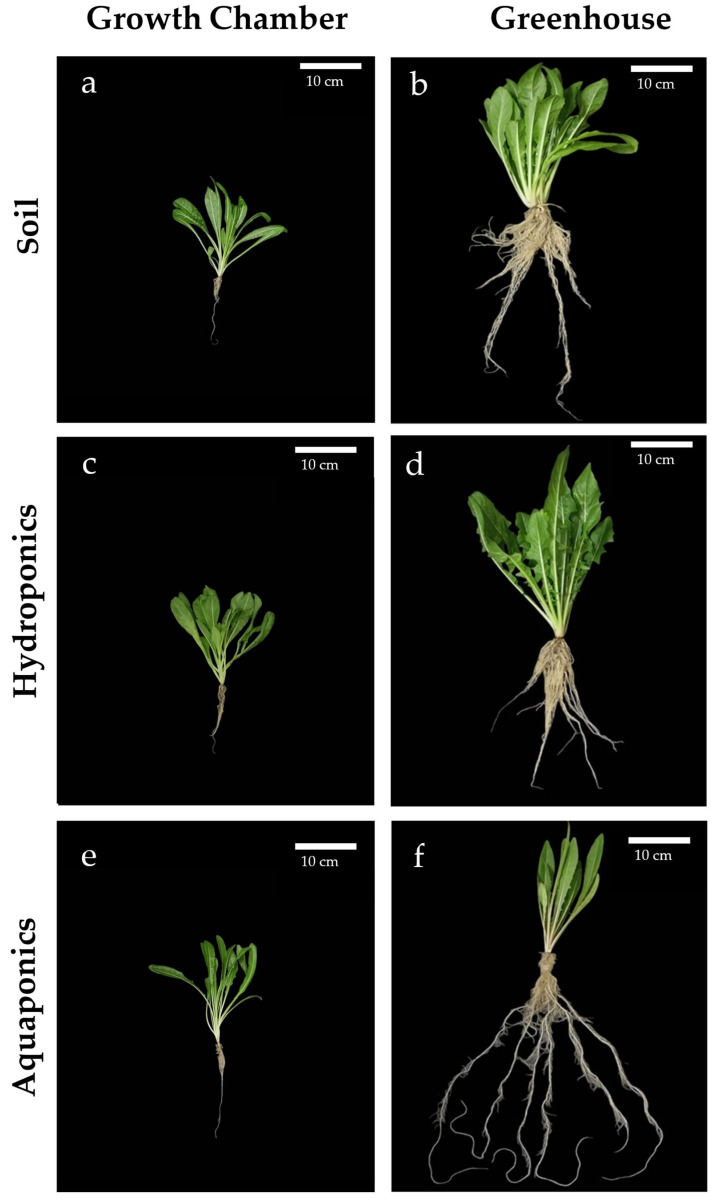
Phenotypic comparison of chicory plants grown in different cultivation environments. Fourty-five-day-old chicory plants were grown in a growth chamber (**a**,**c**,**e**) or in a greenhouse (**b**,**d**,**f**) under soil (**a**,**b**), hydroponic (**c**,**d**), and aquaponic (**e**,**f**) conditions. Scale bars: 10 cm.

**Figure 2:**
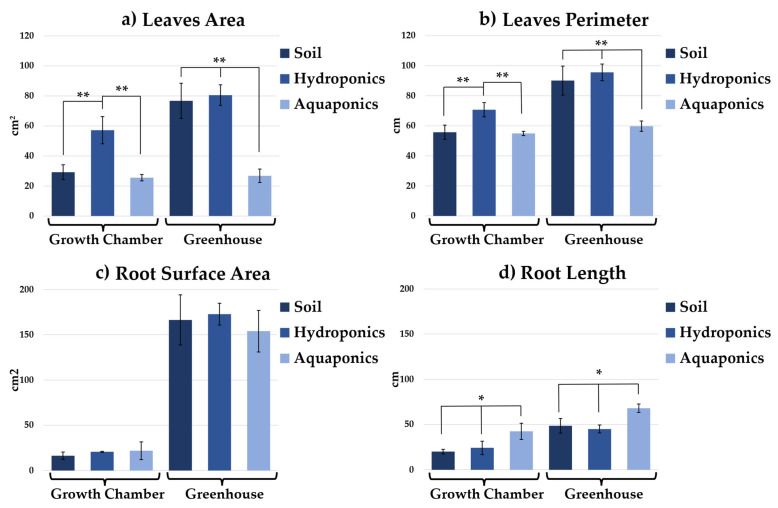
Leaves area (cm^2^) (**a**), perimeter (cm) (**b**), root surface area (cm^2^) (**c**) and longest (cm) (**d**) of chicory grown in soil, hydroponics, and aquaponics under two the different growth environments. Significant differences among cultivation systems within each environment are indicated as *p* < 0.05 (*) and *p* < 0.01 (**), according to one-way ANOVA followed by Tukey’s post hoc test.

Phenotypic comparison revealed that both the cultivation system and the growth environment significantly influenced chicory morphology (Figure 1). Morphometric analyses (Figure 2) confirmed that greenhouse cultivation significantly increased leaf area and perimeter relative to the growth chamber across all systems. Within the growth chamber, soil- and aquaponic-grown plants (Figure 1a,e) exhibited comparable growth (leaf area 29.21 ± 4.95 cm^2^ and 25.52 ± 2.14 cm^2^; perimeter 55.68 ± 4.72 cm and 54.96 ± 1.42 cm, respectively), whereas hydroponically grown plants (Figure 1c) developed significantly larger leaves (leaf area 57.16 ± 9.09 cm^2^; perimeter 70.64 ± 4.73 cm). In the greenhouse, soil- and hydroponic-grown plants (Figure 1b,d) reached the greatest vigor and leaf size (leaf area 76.69 ± 11.77 cm^2^ and 80.50 ± 6.86 cm^2^; perimeter 90.14 ± 9.66 cm and 95.60 ± 5.52 cm), whereas aquaponic leaves were smaller, showing a 26.7% reduction in leaf area relative to hydroponics (Figure 1f).

Root morphometry also varied with environment and cultivation system (Figure 2). Although root surface area did not differ significantly among treatments; greenhouse-grown plants displayed markedly higher average values (153.97 ± 23.02 cm^2^ to 172.9 ± 12.11 cm^2^) than growth chamber plants (16.3 ± 4.1 cm^2^ to 21.9 ± 9.8 cm^2^), suggesting more extensive and uniform root biomass. Maximum root length differed primarily in aquaponic system, reaching 42.5 ± 9.0 cm in the growth chamber and 68.2 ± 4.7 cm in the greenhouse, indicating adaptive elongation in response to nutrient solution characteristics. Soil- and hydroponic-grown roots remained shorter in both environments. Root length in the growth chamber was 20.1 ± 2.6 cm in soil and 24.3 ± 7.4 cm in hydroponics, while in the greenhouse it reached 48.8 ± 8.1 cm and 45.1 ± 4.4 cm, respectively. Under all experimental conditions, plants appeared healthy, with no symptoms of stress such as leaf yellowing, wilting, or morphological abnormalities, and with no signs of root rot, ensuring maintained consumer appeal.

**Figure 3:**
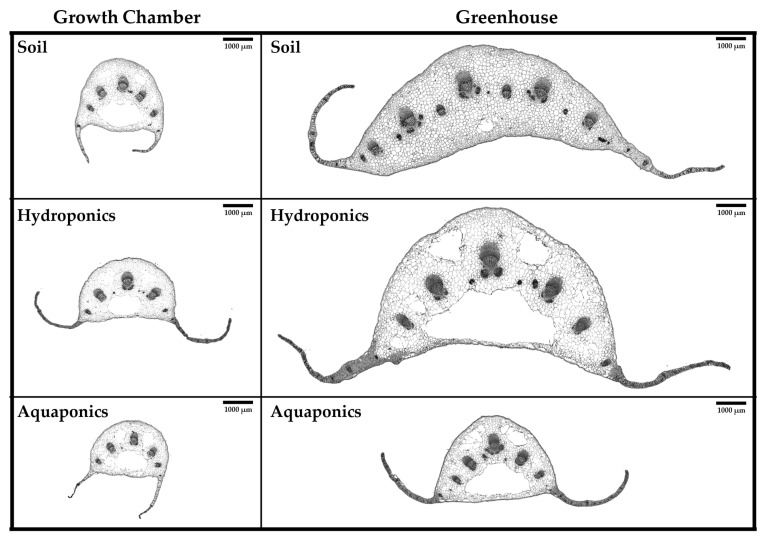
Bright field images of transverse sections of the basal leaf central vein taken from chicory leaves of 45-day old grown in the different experimental conditions: soil, hydroponics, and aquaponics under two growth environments: growth chamber and greenhouse. Scale bars = 1000 μm.

Leaf cross-sections corroborated macroscopic observations (Figure 3). Dorsoventral vascular bundle organization was consistent across conditions, and parenchyma cell exhibited a progressive increase in size from leaf margins toward the center of the lamina.

In the growth chamber, transverse leaf sections exhibited reduced dimensions and consistently displayed five well-differentiated vascular bundles. Soil- and hydroponic-grown leaves had larger central vascular bundles (0.16 ± 0.005 mm^2^ and 0.17 ± 0.008 mm^2^, respectively) than aquaponic leaves (0.08 ± 0.004 mm^2^; Table S1a). Growth chamber leaves also exhibited three lysigenous cavities: one central and two interveinal cavities of variable size, with total areas of 0.97 ± 0.021 mm^2^ (soil), 0.99 ± 0.029 mm^2^ (hydroponics), and 1.42 ± 0.062 mm^2^ (aquaponics; Table S1b).

Greenhouse leaves were broader, with a larger mesophyll and several vascular bundles of variable dimensions. All samples displayed five larger, well-developed vascular bundles. The central vascular bundle area was 0.36 ± 0.002 mm^2^ in soil-grown plants, 0.54 ± 0.014 mm^2^ in hydroponic plants, and 0.19 ± 0.001 mm^2^ in aquaponic plants (Table S1a). Multiple lysigenous cavities appeared in hydroponic and aquaponic leaves, whereas soil-grown leaves had compact parenchyma with a single small cavity.

The total lysigenous cavity areas were 0.17 ± 0.047 mm^2^ in soil-grown plants, 7.63 ± 0.251 mm^2^ in hydroponic plants, and 2.31 ± 0.067 mm^2^ in aquaponic plants (Table S1b). These variations are likely related to plant adaptation to growing conditions and leaf size. Indeed, the percentage ratio between total lysigenous cavity area and central vein area indicated that, in the growth chamber, soil-grown plants exhibited a ratio of 22.13%, hydroponic plants 14.37%, and aquaponic plants 22.24%, whereas in the greenhouse the corresponding values were 0.80%, 24.65%, and 22.67%, respectively.

Overall, anatomical analysis highlighted marked plasticity of lysigenous cavities in response to both growth environment and cultivation system. Leaves grown in the growth chamber showed a consistent pattern, while greenhouse conditions amplified the differences: hydroponic and aquaponic cultivation produced larger and more numerous cavities, while plants grown in soil maintained a compact parenchyma. These results suggest that cavity formation does not depend solely on size, but reflects adaptive responses to environmental and cultural conditions, potentially linked to stress perception or enhanced secondary metabolism.

### 2.2. Edible Biomass Production

**Figure 4:**
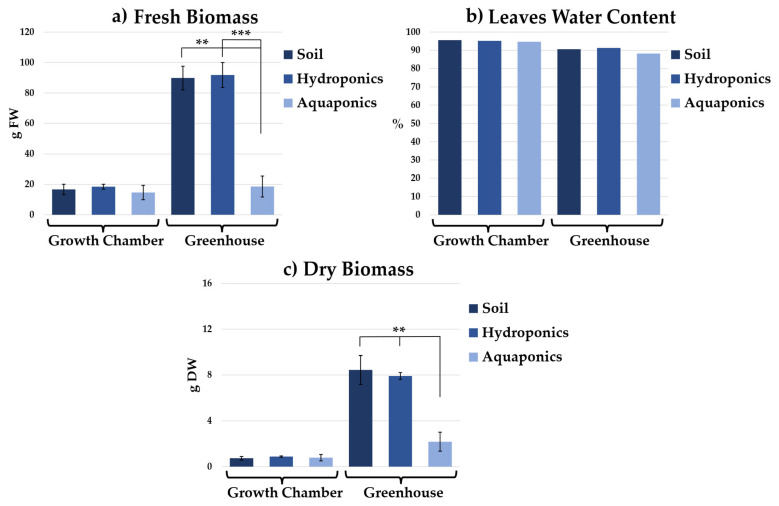
Fresh biomass (g FW) (**a**), leaf water content (%) (**b**), and dry biomass (g DW) (**c**) of chicory grown in soil, hydroponics, and aquaponics under two growth environments (Growth chamber and Greenhouse). Significant differences among cultivation systems within each environment are indicated as and *p* < 0.01 (**) and *p* < 0.001 (***) according to one-way ANOVA followed by Tukey’s post hoc test.

Fresh and dry edible biomass measurements mirrored morphometric trends (Figure 4). Greenhouse-grown plants, particularly in soil and hydroponics, accumulated significantly more biomass than those in the growth chamber, while aquaponic biomass remained lower. The highest fresh biomass, expressed as Fresh Weight (FW) (Figure 4a), was recorded in soil-grown and hydroponically grown greenhouse plants (89.9 ± 7.8 g FW and 91.8 ± 8.2 g FW, respectively), compared with 18.56 ± 4.88 g FW in aquaponically grown plants. In contrast, plants cultivated in the growth chamber exhibited markedly lower fresh biomass values, ranging from 14.7 ± 4.7 g FW to 18.5 ± 1.7 g FW across cultivation systems. Leaf water content was high across all cultivation systems in the two environmental conditions (growth chamber: 94.68–95.59%; greenhouse: 88.28–91.31%) (Figure 4b) in fact, a similar trend to that observed for fresh biomass was also found for dry biomass (Figure 4c). In the growth chamber, dry biomass, expressed as Dry Weight (DW), remained low and similar across all cultivation systems (0.88 ± 0.05 g DW in hydroponics, 0.73 ± 0.15 g DW in soil, and 0.78 ± 0.28 g DW in aquaponics). In the greenhouse, soil-grown and hydroponically grown plants reached comparable DW values (8.4 ± 1.3 g and 7.4 ± 1.1 g respectively), whereas aquaponically grown plants accumulated substantially less dry biomass (2.2 ± 0.8 g DW).

### 2.3. Biochemical Characterization

All biochemical analyses were conducted on leaves of 45-day-old chicory plants grown under soil, hydroponic, and aquaponic systems in the two different environmental conditions. Only leaves were analyzed, as they represent the edible portion of the chicory plant.

#### 2.3.1. Products of Primary Metabolism

Analysis of soluble and insoluble polysaccharides in chicory leaves revealed significant differences in the soluble fraction among experimental conditions. Leaves of soil-grown plants in the growth chamber contained 3.24 ± 0.6 mg/g FW, whereas aquaponically grown plants in the greenhouse accumulated the highest levels of soluble polysaccharides, reaching 14.25 ± 3.2 mg/g FW, a value significantly higher than all other treatments. Insoluble polysaccharides were also present in varying amounts, with a decrease in soilless systems in growth chambers (2.04 ± 0.06 in hydroponincs and 2.23 ± 0.07 in aquaponics) and a marked increase in the same cultivation methods in greenhouses (1.88 ± 0.09 in hydroponincs and 2.60 ± 0.07 in aquaponics) (Table 1).

Total protein content in chicory leaves showed significant differences in the growth chamber. Hydroponically grown plants contained 4.31 ± 0.14 mg/g FW, aquaponically grown plants 3.94 ± 0.41 mg/g FW, and soil-grown plants 2.71 ± 0.21 mg/g FW. No significant differences were observed under greenhouse conditions, where values ranged from 3.91 ± 0.06 mg/g FW to 4.62 ± 0.17 mg/g FW.

Lipid content in chicory leaves was consistently higher in soilless systems than in soil-based cultivation. In the growth chamber, soil-grown plants contained 45.0 ± 2.35 mg/g FW, whereas hydroponic and aquaponic plants reached 56.43 ± 6.19 mg/g FW and 55.57 ± 1.72 mg/g FW, respectively. A similar pattern was observed in the greenhouse, with values of 51.53 ± 0.59 mg/g FW in soil, 63.77 ± 4.86 mg/g FW in hydroponics, and 62.83 ± 2.40 mg/g FW in aquaponics. These results indicate that soilless cultivation enhances lipid accumulation relative to conventional soil cultivation in both growth environments (Table 1).

#### 2.3.2. Chlorophyll and Carotenoid Quantification

Analysis of photosynthetic pigments in chicory leaves revealed distinct differences related to both growth environment and cultivation system (Table 2). Chlorophyll a content was significantly highest in hydroponically grown plants in the growth chamber (1.42 ± 0.07 mg/g FW), followed by aquaponic (1.15 ± 0.08 mg/g FW) and soil-grown plants (1.14 ± 0.09 mg/g FW). Under greenhouse conditions, Chlorophyll a levels decreased overall; however, hydroponically grown plants maintained relatively high concentrations (1.34 ± 0.12 mg/g FW), while soil-grown (0.98 ± 0.11 mg/g FW) and aquaponic-grown plants (0.89 ± 0.10 mg/g FW) exhibited further reductions.

Chlorophyll b levels showed no significant differences among cultivation systems in the growth chamber (Table 2). In the greenhouse, Chlorophyll b exhibited the highest concentration in hydroponically grown plants (0.71 ± 0.12 mg/g FW).

The carotenoid content did not show statistically significant variations between cultivation systems or growth environments (Table 2).

The chlorophyll a/b ratio (Table 2), an indicator of photosynthetic efficiency and light acclimation, displayed contrasting patterns between environments. In the growth chamber, the lowest ratios were observed in soil-grown (2.12) and aquaponic-grown plants (2.16), whereas hydroponically grown plants exhibited a significantly higher ratio (2.75). Conversely, in the greenhouse, the highest ratio was observed in soil-grown plants (3.01), while hydroponic and aquaponic plants showed lower values (1.87 and 2.00, respectively).

#### 2.3.3. Secondary Metabolites and Antioxidant Activity

**Figure 5:**
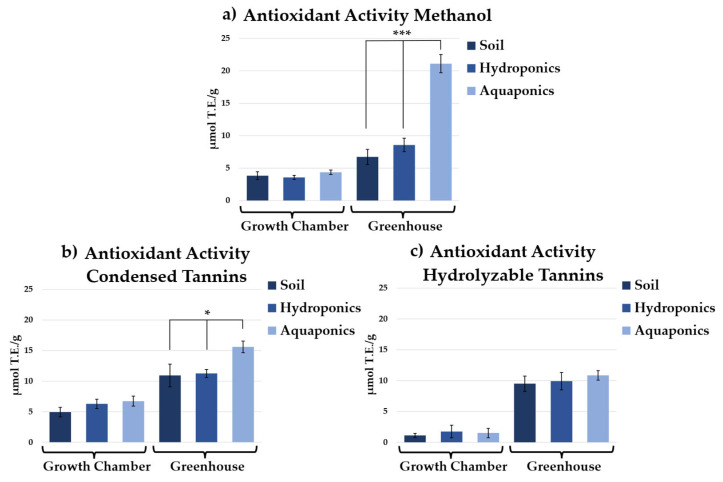
Antioxidant activity of chicory extracts obtained with methanol (**a**), condensed tannins (**b**), and hydrolyzable tannins (**c**) amount in plants grown in soil, hydroponics, and aquaponics under two growth environments. Values are expressed as μmol Trolox Equivalents (TE) per g FW. Significant differences among cultivation systems within each environment are indicated as *p* < 0.05 (*) and *p* < 0.001 (***) according to one-way ANOVA followed by Tukey’s post hoc test.

Antioxidant activity of different fractions extracted from chicory leaves varied markedly depending on growth environment and cultivation system (Figure 5). Methanolic extracts (Figure 5a) showed no significant differences among all plants grown in the growth chamber (3.83 ± 0.6 μmol TE/g FW in soil, 3.56 ± 0.3 μmol TE/g FW in hydroponics, and 4.35 ± 0.35 μmol TE/g FW in aquaponics). In contrast, under greenhouse conditions, a substantial increase in antioxidant activity was observed in aquaponically grown plants (21.10 ± 1.40 μmol TE/g FW). This value was approximately 2.5-fold higher than that of hydroponically grown plants (8.57 ± 1.04 μmol TE/g FW) and more than threefold higher than that of soil-grown plants (6.73 ± 1.18 μmol TE/g FW), highlighting the combined influence of cultivation method and environmental conditions on antioxidant accumulation.

A similar trend was observed for condensed and hydrolyzable tannins. Within the same environment, significant differences were detected only for condensed tannins in greenhouse-grown aquaponic plants, which reached 15.61 ± 0.97 μmol TE/g FW (Figure 5b). Although there was a clear increase in hydrolysable tannins in the greenhouse compared to those recorded in the growth chamber, no significant differences were observed within the two groups in relation to the different cultivation systems (Figure 5c).

**Figure 6:**
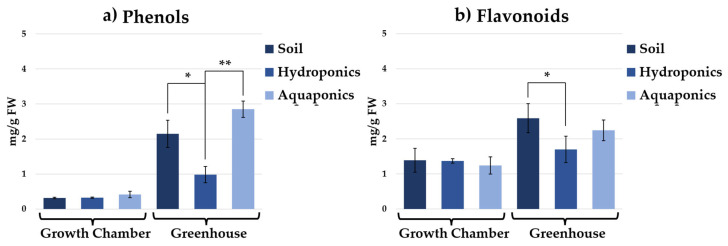
Concentration of phenols (mg/g FW) (**a**) and flavonoids (mg/g FW) (**b**) in chicory plants grown in soil, hydroponics, and aquaponics under two growth environments. Significant differences among cultivation systems within each environment are indicated as *p* < 0.05 (*) and *p* < 0.01 (**) according to one-way ANOVA followed by Tukey’s post hoc test.

Total phenolic and flavonoid contents further followed these patterns (Figure 6). In the growth chamber, no significant differences were detected among cultivation systems. In the greenhouse, differently aquaponically grown plants exhibited the highest total phenolic content (2.85 ± 0.23 mg/g FW), followed by soil-grown plants (2.15 ± 0.39 mg/g FW) (Figure 6a). In greenhouse, flavonoid content (Figure 6b) was highest in soil-grown plants (2.59 ± 0.41 mg/g FW), indicating that the accumulation of specific secondary metabolites depends on both cultivation system and environmental conditions.

### 2.4. Abiotic Conditions

**Figure 7:**
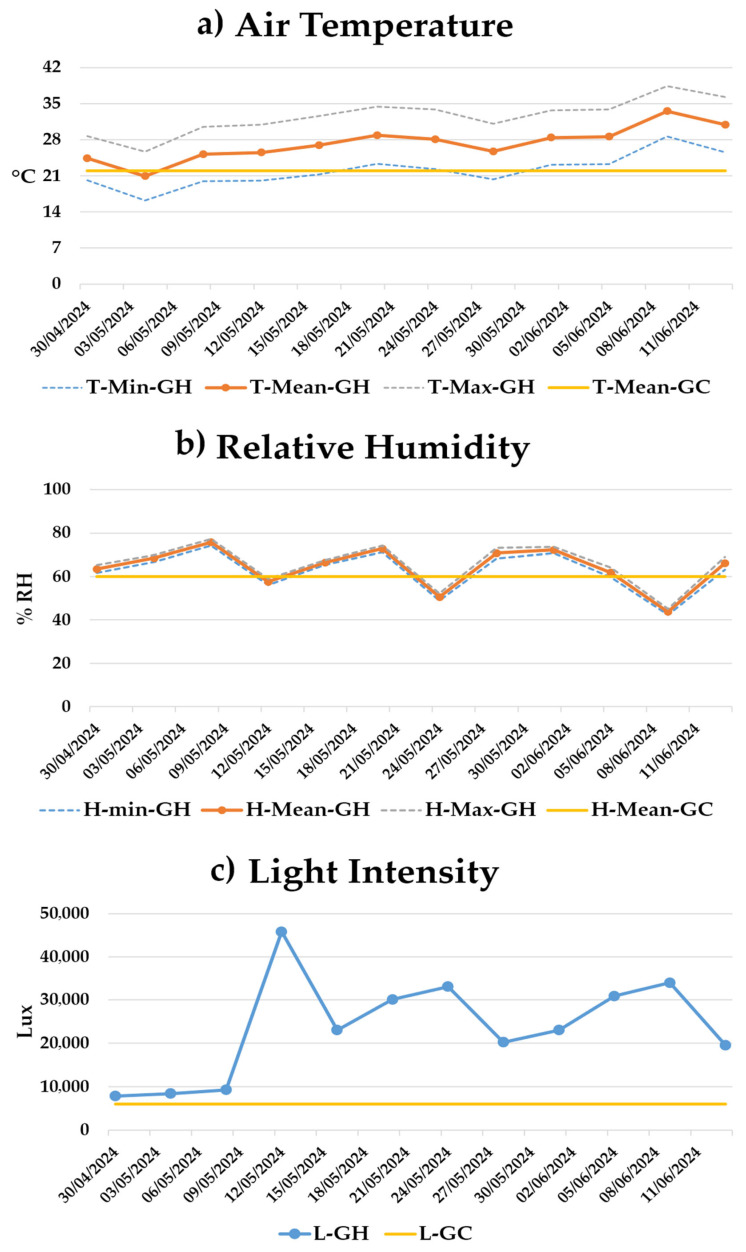
Trend of environmental conditions in greenhouse (GH) and growth chamber (GC): air temperature (**a**), relative humidity (**b**), and light intensity (**c**).

Environmental monitoring in the greenhouse confirmed highly dynamic growing conditions throughout the experimental period (Figure 7). Daily minimum temperatures ranged from 16.2 °C to 28.7 °C, while maximum temperatures varied from 25.7 °C to 38.4 °C, exposing plants to frequent midday heat peaks (Figure 7a). The average temperature during the growth period was 27 °C.

Relative humidity fluctuated considerably, with average values ranging from 43.8% to 75.8%. These variations reflect the interaction between seasonal temperature increases and greenhouse ventilation, with an overall average relative humidity of 64.2% during the growth period (Figure 7b).

Light intensity was the most variable parameter, ranging from 7.8 to 45.8 kLux, with exceptionally high peaks on clear days (Figure 7c).

Overall, these environmental data indicate that greenhouse-grown plants were exposed to more variable and intense conditions, including higher light intensity and fluctuating temperature and humidity, compared with the stable environmental conditions of growth chamber. These conditions likely contributed to the enhanced synthesis of secondary metabolites and antioxidant compounds observed in greenhouse-grown chicory.

## 3. Discussion

This comparative study on chicory plants grown in soil, hydroponics, and aquaponics reveals that growing environment conditions strongly influence plant development, while cultivation systems exert distinct, system-specific effects. Under the experimental conditions tested, greenhouse-grown plants consistently exhibited greater vigor than those cultivated in the growth chamber across all measured parameters. This enhanced performance is likely attributable to higher light intensity and more favorable temperature fluctuations in the greenhouse environment. Since this trend was also observed in plants grown under the same cultivation system in both growth environments, the differences in plant performance can be likely primarily ascribed to abiotic factors, namely variations in temperature, humidity, and light intensity characteristic of greenhouse conditions.

Integration of environmental data (Figure 7) indicates that the variability in temperature (16–38 °C), relative humidity (44–76%), and light intensity (7.8–45.8 kLux) in the greenhouse promoted leaf expansion, mesophyll development, vascular differentiation, and the accumulation of secondary metabolites, particularly phenolics and antioxidants in aquaponically grown plants. Conversely, the stable and controlled conditions of the growth chamber constrained these adaptive responses, resulting in smaller leaves and lower stress-induced metabolite production. These findings highlight that morpho-physiological plasticity in chicory is strongly influenced by environmental variability, often more than by the cultivation system itself. The role of the growing environment extends beyond plant performance and also affects the overall sustainability of production systems, as noted by [24].

Within each environment, hydroponic plants generally exhibited the most vigorous growth, consistent with previous reports on lettuce and other leafy vegetables, where nutrient stability, optimal water supply, and enhanced root-zone aeration in hydroponics facilitated leaf expansion [25]. Soil-grown plants showed slightly lower, yet comparable performance, whereas aquaponically grown plants consistently displayed growth limitations and adaptive stress responses. Specifically, aquaponic plants produced smaller leaves and pronounced root elongation, consistent with known nutrient and oxygen dynamics limitations in aquaponic systems [23,26].

The pronounced root elongation observed in aquaponic plants is a well-documented morphological response to nutrient or oxygen limitation, enabling plants to explore a larger substrate volume and improve resource uptake efficiency [27,28]. In the decoupled aquaponic system used here, suboptimal nutrient dynamics—primarily caused by organic matter accumulation and insufficient pH regulation—likely triggered this compensatory root elongation, reflecting an adaptive strategy rather than enhanced growth. pH is a crucial factor for nutrient availability, and variations in pH can result in the precipitation of salts in nutrient solutions, affecting nutrient absorption by plants.

Microscopic observations of transverse leaf sections reinforced the morphometric findings. Greenhouse-grown leaves displayed thicker laminae, more voluminous mesophylls, and larger vascular bundles than leaves from the growth chamber, reflecting environmental acclimation. Higher light intensity and fluctuating temperature regimes in greenhouse conditions stimulate tissue differentiation and vascular development [29,30]. While the size of individual xylem and phloem vessels remained unchanged, the number of vessels increased in hydroponically grown plants, which also exhibited multiple vascular complexes in the midrib. This organization was absent in other samples, where only less developed bundles were adjacent to the main vascular bundle.

As previously reported by [31], chicory leaves can develop lysigenous cavities of variable size depending on the cultivar, a feature enhanced under water stress [32]. In this study, only soil-grown greenhouse leaves showed small lysigenous cavities, whereas all other samples exhibited larger cavities, reflecting both environmental conditions and cultivation-induced stress responses.

Edible biomass production corroborated the morphological and anatomical trends. Fresh biomass was consistently higher in greenhouse-grown hydroponic and soil-based plants, reflecting greater leaf expansion and tissue hydration. Plants in these systems achieved similar developmental outcomes in terms of yield but exhibited distinct parenchymatic and structural architectures tailored to their growth conditions [33,34,35]. Aquaponics produced the lowest biomass in the greenhouse, confirming growth limitations and aligning with anatomical evidence of reduced vessel and mesophyll development [36]. In the growth chamber, biomass production was uniformly lower across all systems, reflecting the strong influence of environmental conditions on cultivation performance.

Biochemical analyses revealed complementary adaptive strategies. Soluble and insoluble carbohydrate accumulation differed among environmental systems, in growth chamber a reduction in the polysaccharides amount in soilless-plant was recorded respect those observed in soil-like cultivation method, while in greenhouse the polysaccharides increased in soilless cultivation with aquaponically grown plants exhibiting the highest levels, indicative of a stress-induced adjustment of primary metabolism. Soluble sugars serve as osmoprotectants, ROS scavengers, and signaling molecules under abiotic stress [37,38]. Their accumulation in aquaponics coincided with reduced growth, reflecting a shift in assimilates from structural development toward protective and regulatory functions.

Protein content peaked in hydroponic and aquaponic plants under growth chamber conditions, likely reflecting higher and more stable availability of assimilable nitrogen [25,35]. Soil-grown plants showed lower protein levels, probably due to limited microbial activity and reduced nitrogen mineralization under fully controlled conditions [39,40]. In the greenhouse, these differences disappeared, as fluctuating temperatures and natural radiation likely enhanced microbial activity and soil nitrogen availability, allowing protein levels to converge across systems, consistent with the larger and more developed root systems observed.

Total lipid content was also higher in hydroponic and aquaponic plants than in soil-grown plants in both environments. This pattern aligns with previous reports that soilless cultivation favors lipid accumulation, likely through continuous nutrient supply and enhanced carbon allocation to lipophilic metabolites and membrane-associated compounds [41].

Photosynthetic pigment analysis revealed higher absolute chlorophyll concentrations in hydroponically grown plants, whereas carotenoid levels remained relatively unchanged across systems. Light regimes differed substantially between environments (growth chamber: ~6000 lux; greenhouse: ~24,000 lux), which likely accounts for most variation in absolute pigment pools. Low-light environments favor accumulation of Chl b, while high-light exposure shifts the balance toward Chl a, reflecting photoadaptive remodeling of the light-harvesting complex [42,43].

The Chl a/b ratio displayed a clear environment × system interaction. In the growth chamber, hydroponic plants had the highest ratio, consistent with a compact antenna under stable LED illumination and abundant nitrogen supply [44,45]. In the greenhouse, soil-grown plants exhibited the highest ratio, indicative of LHCII downsizing under strong and fluctuating irradiance, whereas soilless plants maintained less contraction of antenna complexes [46,47]. Carotenoid stability suggests that xanthophyll cycle-based photoprotection sufficed without additional pigment synthesis [48].

Regarding secondary metabolism profile, significant differences were evident primarily in greenhouse aquaponic and soil-grown plants. In aquaponics, high antioxidant activity in methanol extracts and condensed tannins (TEAC assay) reflects elevated stress-induced phenolic accumulation, consistent with the inducible protective role of proanthocyanidins under abiotic stress [49,50,51]. Conversely, flavonoid accumulation was more pronounced in soil-grown plants, consistent with rhizosphere-mediated modulation of secondary metabolism rather than stress induction [40,52].

Environmental factors are well known to influence secondary metabolism across cultivation systems. Several studies have shown that pH, light, and temperature significantly affect plant growth and secondary metabolite production [53,54]. Temperature modulates key physiological processes such as respiration, photosynthesis, and transpiration. For example, increasing temperature enhances yield and photosynthetic rate in rice but reduces photosynthesis in cotton [54]. Light spectrum also plays a pivotal role in secondary metabolite synthesis; red light increases phenolic content in *Crepidiastrum denticulatum* [55] and stimulates amaranthine and vindoline accumulation in *Catharanthus roseus* L. [56].

Overall, these findings reveal two complementary adaptive strategies: aquaponics enhances generalized stress-related phenolic metabolism, whereas soil cultivation promotes flavonoid biosynthesis through rhizosphere interactions. This duality underscores the sensitivity of chicory secondary metabolism to environmental and cultivation factors, highlighting phenolics as both stress indicators and mediators of plant-environment interactions.

From an agronomic perspective, hydroponics offers consistent biomass and leaf quality, supporting stable year-round production. Aquaponics, while attractive for circular resource use, requires careful nutrient and pH management to prevent growth limitations and unwanted stress responses. The pronounced influence of environmental variability emphasizes the importance of supplemental lighting, climate buffering, and real-time monitoring in greenhouse production. Collectively, these insights can help optimize input efficiency, crop quality, and system stability across different cultivation setups.

## 4. Materials and Methods

### 4.1. Plant Material and Growth Conditions

*Cichorium intybus* L. seeds were initially soaked in running tap water for 2–3 h and then sown into seedbeds placed within a controlled growth chamber. The chamber was maintained at a constant temperature of 22 °C, relative humidity of 60%, and artificial lighting of 6000 lux (ranging from 3000 to 20,000 lux), under a 16 h light and 8 h dark photoperiod. After four weeks, seedlings were selected and transferred to two cultivation environments: a growth chamber located in the biology section of the University of Salento and a greenhouse located at the Botanical Garden of the University of Salento (Latitude 40.3450° N, Longitude 18.1231° E).

### 4.2. Cultivation Systems

A total of 54 uniform seedlings were selected for the experiment. Each environment hosted 27 plants, evenly distributed among three cultivation systems: soil-based, hydroponics, and aquaponics. Consequently, 9 plants were assigned to each treatment, with each plant serving as an independent biological replicate. In this study, soil cultivation refers to the cultivation of plants in pots filled with a solid organic substrate. Although cultivation in containers with substrate is technically classified as a soilless system according to [57], the term soil was used to refer to a soil-like cultivation system with a solid rooting medium that did not receive fertigation during the entire crop cycle. Plants grown in soil were placed in 4.5 L plastic pots filled with a standard organic substrate (Turco Silvestro^®^, TS-ATOMIC–ULTIMATE^®^, Albenga, SV, Italy) composed of acid peat (45%), coconut fiber (25%), pumice (15%), mature manure (10%), dolomite (3%), organic nitrogen fertilizer (1.5%), and mycorrhizal inoculum (0.5%). The properties of the substrate were: pH 6.5, bulk density 220 kg/m^3^, total porosity 90% (*v*/*v*), air capacity 25% (*v*/*v*), water retention at pF 1 65% (*v*/*v*), EC 0.30 dS/m^−1^, organic matter 68% (d.w.) and organic C 34% (d.w.). Irrigation was carried out every three days: 800 mL/pot in the greenhouse and 100 mL/pot in the growth chamber, taking into account the different evapotranspiration requirements. The soilless systems (hydroponics and aquaponics) were established using identical plastic containers measuring 31 cm × 38 cm × 22.5 cm with a working volume of 18 L. In both systems, plants were supported by floating platforms, and perlite was used as the root substrate. Both systems operated in a closed-loop configuration throughout the 45-day growth period, with nutrient solutions applied only once at the start of the experiment.

In the hydroponic system, a commercial nutrient solution (Advanced Nutrients^®^ pH Perfect^®^ Grow, Micro, Bloom, West Hollywood, CA, USA) was used. The concentration of macronutrients and micronutrients was as follows: nitrate (NO_3_^−^) 2.26 mmol L^−1^, ammonium (NH_4_^+^) 0.59 mmol L^−1^, potassium (K^+^) 1.70 mmol L^−1^, urea 1.83 mmol L^−1^, phosphate (H_2_PO_4_^−^) 0.42 mmol L^−1^, and sulfate (SO_4_^2−^) 0.50 mmol L^−1^, calcium (Ca^2+^) 0.86 mmol L^−1^ and magnesium (Mg^2+^) 0.10 mmol L^−1^. Micronutrients included iron (Fe) 17.9 µmol L^−1^, manganese (Mn) 7.28 µmol L^−1^, and boron (B) 37.0 µmol L^−1^. The chemical-physical parameters of the hydroponic nutrient solution remained stable throughout the experiment, with a pH of 6.5.

In the aquaponic system, nutrient supply was provided by wastewater from a recirculating aquaculture system (RAS) housing tilapia. The concentrations in the aquaponic solution included ammonia (NH_3_) at 1.7 × 10^−5^ mol/L, nitrate (NO_3_^−^) at 3.29 × 10^−4^ mol/L, nitrite (NO_2_^−^) at 4.35 × 10^−7^ mol/L, potassium (K^+^) at 2.37 × 10^−3^ mol/L, and phosphorus at 2.24 × 10^−7^ mol/L. Micronutrient concentrations were calcium (Ca) at 4.77 × 10^−3^ mol/L, magnesium (Mg) at 8.48 × 10^−4^ mol/L, iron (Fe) at 2.36 × 10^−5^ mol/L, sulfates (SO_4_^2−^) at 2.9 × 10^−3^ mol/L, chloride (Cl^−^) at 1.11 × 10^−3^ mol/L, zinc (Zn) at 3.06 × 10^−7^ mol/L, copper (Cu) at 6.93 × 10^−7^ mol/L, and manganese (Mn) at 2.11 × 10^−6^ mol/L. The average pH in this system was 8.0.

Environmental conditions for the soilless systems were consistent with their respective locations. In the growth chamber, plants growth continued under the same controlled conditions used for germination: 22 °C, 60% relative humidity, 6000 lux light intensity, and a 16/8 h light/dark photoperiod. In the greenhouse, conditions were semi-controlled and corresponded to the optimal seasonal growth period. For environmental monitoring, daily temperature and humidity were measured using a thermo-hygrometer (ThermoPro TP50, Atlanta, GA, USA with an accuracy of ±0.1 °C and a measurement range between −20 °C and +60 °C), positioned adjacent to the growth systems.

### 4.3. Histological Analysis

Chicory plants, grown for 45 days under the four experimental conditions, were used for histological purposes. A 5 mm-thick leaf portion of an isolated leaf, for each treatment, was cut with a razor blade starting at 5 cm from the base and immediately fixed in cold (4 °C) FAA solution (50% ethanol, 0.5% glacial acetic acid, 10% formaldehyde 37–40%, water to final volume) overnight. The fixed specimens were dehydrated and then embedded in paraffin. Paraffin blocks were sectioned to a thickness of 15 μm with a LEICA RM2155 (Leica Microsystems, Wetzlar, Germany) microtome as described in [58]. Sections were mounted on slides and deparaffinized using Xylene, transitioned in Xylene/Ethanol (1:1 *v*/*v*), and then dehydrated with Ethanol. Images were acquired with a 10x objective and a Zeiss LSM900 microscope (Carl Zeiss Microscopy, Jena, Germany). For each sample, a defined number of 2D confocal sections (tiles) were scanned using a motorized stage and automatically mounted with the Tile tool of the Zen Blue Edition (Carl Zeiss Microscopy GmbH, Jena, Germany) program to obtain the bright-field image of the entire sample. Leaf and root morphometry were analyzed using ImageJ (version 1.53e; NIH, Bethesda, MD, USA) on calibrated photographs. Leaf area (cm^2^) and perimeter (cm) were measured by tracing the entire leaf margin, capturing morphological variations including lobation and margin complexity. For roots, surface area (cm^2^) and maximum length (cm) were determined.

### 4.4. Evaluation of Biomass

Biomass production was assessed by measuring the fresh weight (FW) and dry weight (DW) of chicory leaves. Basal leaves of the rosette, representing the edible biomass, were harvested, immediately weighed for fresh weight using a precision balance (Mettler Toledo PC 440, Columbus, OH, USA), and photographed for morphological analysis. Leaf and root morphometry (e.g., major and minor axes, area, and perimeter) were analyzed using ImageJ (version 1.53e; National Institutes of Health, Bethesda, MD, USA), following established protocols [59]. To determine the dry weight, 250 mg of leaf tissue was dried at 60 °C for 1 week, or until the biomass weight stabilized. The water content of chicory leaves was calculated as the difference between fresh weight and dry weight [32].

### 4.5. Evaluation of Polysaccharides, Proteins and Lipids

The extraction of polysaccharides from chicory leaves was performed according to the method described by [60]. Prior to extraction, samples underwent a pretreatment with 70% ethanol to remove soluble sugars present in the plant tissue. Following the extraction of polysaccharides, their quantification was conducted using a colorimetric technique described by [61].

For protein analysis, total protein extraction was performed following the protocol described by [31], and the quantification was performed using the [62] method.

Total lipids were extracted and quantified using a modified [63] method. Fresh leaves were homogenized in liquid nitrogen and treated with 3 mL chloroform:methanol (1:2, *v*/*v*), followed by sonication for 5 min at room temperature. After centrifugation (3000 rpm, 5 min, 22 °C), the supernatant was collected and washed again with the same solvent mixture. Combined supernatants were mixed with 2 mL chloroform and 1.2 mL of 1% KCl, vortexed, and centrifuged under the same conditions. The lower organic phase was recovered and dried at 40 °C for 24 h. Lipid content was then quantified using an analytical balance (Mettler Toledo PC 440, Columbus, OH, USA).

### 4.6. Evaluation of Chlorophyll and Carotenoid Content, Flavonoids and Soluble Phenols

To assess food quality in terms of water stress, chlorophyll (a, b, total) and carotenoid concentrations were determined spectrophotometrically using a Shimadzu^®^ UV-2600 instrument (Kyoto, Japan), following the methods of [64]. The results were expressed as nmol/g FW [32].

Flavonoids were extracted from the plant material following the method described by [64], which involves solvent-based extraction under controlled conditions. Quantification of total flavonoid content was then carried out using a combined approach, following both the spectrophotometric procedure outlined by [65] and the colorimetric method originally developed by [66].

### 4.7. Evaluation of Antioxidant Activity

The sequential extraction of water-soluble and fat-soluble antioxidants was performed following a modified version of the method described by [67]. Aliquots of 0.1 g of freeze-dried chicory leaves were subjected to successive extractions with 1.2 mL each of methanol, acetone, and hexane, under constant stirring at 300 rpm for 1 h. Each extraction step was followed by centrifugation at 2500× *g* for 10 min. After collecting the final supernatants, the residual pellets were used for the extraction of bound tannins. Condensed tannins were extracted using 1 mL of a butanol/hydrochloric acid solution (95:5, *v*/*v*), incubated at 100 °C for 3 h, and then centrifuged at 2500× *g* for 10 min. Hydrolysable tannins were extracted with 1 mL of a methanol/concentrated sulfuric acid solution (90.9:9.1, *v*/*v*), incubated at 85 °C for 20 h, followed by centrifugation at 2500× *g* for 10 min. The antioxidant activities of the hydrophilic and lipophilic fractions were evaluated on the collected supernatants using the Trolox Equivalent Antioxidant Capacity (TEAC) assay, as described by [60]. Results were expressed as mmol Trolox equivalents (TEs) per g FW.

### 4.8. Statistical Analysis

Statistical analyses were conducted separately for each environment (growth chamber and greenhouse) in order to account for their distinct environmental conditions and rigorously evaluate the effects of cultivation systems (soil, hydroponics, aquaponics) without confounding them with environmental variability. Statistical significance was determined using one-way ANOVA with PAST software4.03 (PAleontological STatistics; University of Oslo, Oslo, Norway) to compare the means of each group. Assumptions of homogeneity of variance were verified using Levene’s test (*p* > 0.05 indicating homogeneity) [68]. If ANOVA indicated significant differences (*p* < 0.05), Tukey’s post hoc tests were performed [69].

## 5. Conclusions

This study confirms that differences in the abiotic conditions of the growth environment are the primary determinants of chicory performance, often modulating or amplifying the effects of the cultivation system. Plants grown under similar environmental conditions exhibited comparable morpho-physiological traits, highlighting the critical role of environmental conditions (temperature, humidity and lux) in maximizing growth and leaf quality. In particular, the stress conditions resulting from high temperatures or changes in humidity and lux can activate defenses or adaptation mechanisms in plants that can have beneficial nutritional effects on the quantity and quality of food products by increasing biomass and metabolite production. Therefore, when developing soil, hydroponic and aquaponic systems, it is important not only to focus on nutrient flows, but also to manipulate environmental conditions to find the right mix to optimize food production quality and quantity in relation to cost and income. Furthermore, this study indicates that, under the same environmental conditions, the choice of cultivation system (soil, aquaponics or hydroponics) can be strategically oriented towards increasing yield and enhancing the functional value of the product, depending on production and nutritional objectives.

Growth chambers, although producing smaller plants, allow for the precise regulation of environmental parameters and technological management, enabling year-round production and potentially earlier harvests for baby leaf crops. Greenhouses provide optimal growth conditions during favorable seasons but require careful management to mitigate environmental variability, including the use of supplemental lighting or temperature control. Among the cultivation systems, hydroponics emerged as the most consistent in promoting leaf expansion, biomass accumulation, and anatomical development, whereas aquaponics revealed the need for refined management of nutrient transfer from fish to plants to ensure stable growth and leaf quality.

Secondary metabolism displayed system-specific responses. Aquaponics promoted phenolic accumulation as part of a generalized stress response, whereas soil-grown plants favored flavonoid biosynthesis mediated by rhizosphere–microbe interactions. These findings suggest practical strategies to enhance the nutraceutical quality of chicory in soilless systems, such as transient modulation of rhizospheric pH or the introduction of beneficial microorganisms, allowing for the stimulation of secondary metabolite production without compromising leaf development or overall quality.

The findings are relevant to the systems and circumstances examined in this study; consequently, additional investigation is required to generalize the findings to other systems or more general circumstances.

## Figures and Tables

**Table 1:** Soluble and insoluble polysaccharides, total protein and lipids content in leaves of chicory grown in soil, hydroponics, and aquaponics under two growth environments. Significant differences among cultivation systems within each environment are indicated as *p* < 0.05 (*) and *p* < 0.01 (**) according to one-way ANOVA followed by Tukey’s post hoc test.

	Growth Chamber			Greenhouse		
	Soil	Hydroponics	Aquaponics	Soil	Hydroponics	Aquaponics
**Soluble** **polysaccharides** **mg/g FW**	3.24 ± 0.60 *	1.56 ± 0.23	1.50 ± 0.24	4.71 ± 0.72	6.54 ± 2.02	14.25 ± 3.20 **
**Insoluble** **polysaccharides** **mg/g FW**	3.46 ± 0.04 *	2.04 ± 0.06	2.23 ± 0.07	1.39 ± 0.08	1.88 ± 0.09 *	2.60 ± 0.07 **
**Protein** **mg/g FW**	2.71 ± 0.21	4.31 ± 0.14 *	3.94 ± 0.41 *	4.22 ± 0.05	3.91 ± 0.06	4.62 ± 0.17
**Lipids** **mg/g FW**	45 ± 2.35	56.43 ± 6.19 *	55.57 ± 1.72 *	51.53 ± 0.59	63.77 ± 4.86 **	62.83 ± 2.40 *

**Table 2:** Chlorophyll a (Chl a), Chlorophyll b (Chl b), Carotenoids content and Chlorophyll a: Chlorophyll b ratio (Chl a:Chl b) in leaves of chicory grown in soil, hydroponics, and aquaponics under two growth environments. Significant differences among cultivation systems within each environment are indicated as *p* < 0.05 (*) and *p* < 0.01 (**) according to one-way ANOVA followed by Tukey’s post hoc test.

	Growth Chamber			Greenhouse		
	Soil	Hydroponics	Aquaponics	Soil	Hydroponics	Aquaponics
**Chl a mg/g FW**	1.14 ± 0.09	1.42 ± 0.07 *	1.15 ± 0.08	0.98 ± 0.11	1.34 ± 0.12 *	0.89 ± 0.10
**Chl b mg/g FW**	0.54 ± 0.06	0.52 ± 0.05	0.53 ± 0.10	0.33 ± 0.09	0.71 ± 0.12 **/*	0.44 ± 0.08
**Carotenoids mg/g FW**	0.26 ± 0.03	0.34 ± 0.03	0.29 ± 0.04	0.28 ± 0.04	0.28 ± 0.08	0.19 ± 0.04
**Chl a:Chl b**	2.12	2.75 *	2.16	3.01 *	1.87	2.00

## Data Availability

All data needed to support the conclusions in the paper are presented in the manuscript and the Supplementary Material.

## Supplementary Materials

The following supporting information can be downloaded at: https://www.mdpi.com/article/10.3390/plants15060974/s1, Table S1. Morphometric parameters of cross sections of the basal leaf of chicory grown in a growth chamber and greenhouse in three systems: soil, hydroponics, and aquaponics. Area of the central bundle (a) and area of the central lysigenous cavity (b). Significant differences among cultivation systems within each environment are indicated as *p* < 0.05 (*), *p* < 0.01 (**) and *p* < 0.001 (***), according to one-way ANOVA followed by Tukey’s post hoc test.
