# Supplementary material for: Environmental and Cultivation Effects on Growth and Phytochemical Profiles of Chicory (Cichorium intybus L.) in Soil, Hydroponics, and Aquaponics
Source: Plants (Basel). 2026 Mar 21;15(6):974. doi: 10.3390/plants15060974 (PMC13029898; doi:10.3390/plants15060974)
Supplement: Supplementary file 1 [file plants-15-00974-s001.zip › plants-4143472-supplementary.pdf]

Table S1. Morphometric parameters of cross sections of the basal leaf of chicory grown in a growth chamber and greenhouse in three systems: soil, hydroponics, and aquaponics. Area of the central bundle (a) and area of the central lysigenous cavity (b). Significant differences among cultivation systems within each environment are indicated as $p < 0.05$ (*), $p < 0.01$ (**) and $p < 0.001$ (***), according to one-way ANOVA followed by Tukey's post hoc test.

	**a) Central vascular bundles area cm²**	
	**Growth Chamber**	**Greenhouse**
Soil	0,16 ± 0,005***	0,36 ± 0,002***
Hydroponic	0,17 ± 0,008***	0,54 ± 0,014***
Aquaponic	0,08 ± 0,004	0,14 ± 0,001
	**b) Lysigenous cavity area cm²**	
	**Growth Chamber**	**Greenhouse**
Soil	1,13 ± 0,061***/*	0,17 ± 0,047
Hydroponic	0,59 ± 0,02	5,85 ± 0,249***
Aquaponic	0,71 ± 0,028***	1,64 ± 0,066***
